# Barriers and Facilitators of Physical Activity Participation in Adolescent Girls: A Systematic Review of Systematic Reviews

**DOI:** 10.3389/fpubh.2021.743935

**Published:** 2021-10-15

**Authors:** Keeva Duffey, Ana Barbosa, Stephen Whiting, Romeu Mendes, Isabel Yordi Aguirre, Antonina Tcymbal, Karim Abu-Omar, Peter Gelius, João Breda

**Affiliations:** ^1^World Health Organization, Regional Office for Europe, Copenhagen, Denmark; ^2^Epidemiology Research Unit–Instituto de Saúde Pública, Universidade do Porto, Porto, Portugal; ^3^Department of Sport Science and Sport, Friedrich–Alexander University Erlangen–Nürnberg, Erlangen, Germany

**Keywords:** adolescence, gender, exercise, physical activity, public health, policy

## Abstract

**Background:** Persistent low physical activity (PA) levels among adolescent girls constitute a public health concern that calls for immediate and evidence-based policy action. This systematic review (SR) aimed to summarize evidence from SRs examining the barriers and facilitators of PA participation in adolescent girls. The objectives were to provide a synthesis of the available evidence and identify key areas for fostering gender-responsive action and policy implications.

**Methods:** A comprehensive search of relevant SR and meta-analyses were performed in PubMed and Cochrane Library, until February 2021. Studies were included if they were SRs or meta-analyses, included adolescent girls aged between 10 and 19 years, and described barriers or facilitators of PA. Two independent authors performed the screening of potentially eligible studies and both assessed the methodological quality of included studies using the AMSTAR 2 tool. The barriers and facilitators were synthesized at environmental, interpersonal, and individual levels.

**Results:** A total of eight SRs were included in the qualitative synthesis. The most frequent barriers identified were the lack of support from peers, family, and teachers, and the lack of time. The most reported facilitators were weight loss, and support from peers, family, and teachers. Key areas for action and policy implementation include an inclusive approach to curriculum development to address gender norms; adequate training of professionals so they have a range of skills to ensure inclusion of adolescent girls; environmental changes in and out of schools to stimulate participation, to allow adolescent girls to be active in a safe and attractive environment; multistakeholder support at local, regional, and national level in incorporating a gender-responsive approach toward PA participation.

**Conclusion:** The results highlight a variety of factors that influences the PA participation of adolescent girls. For the attainment of effective policies that increase PA levels in adolescent girls, it is essential to engage several stakeholders at different levels in incorporating a gender-responsive approach toward PA participation.

**Systematic Review Registration:** PROSPERO, identifier: CRD42020204023.

## Introduction

Regular physical activity (PA) has well-known positive effects on the prevention and control of non-communicable diseases, such as cardiovascular diseases, cancer, diabetes, and depression (1–4), as well as reduced overall mortality and risk of premature death (5). In children and adolescents, adequate PA also provides benefits to cognitive development, motor skills, self-esteem, social integration (6), musculoskeletal health (7) academic achievement (8), and overall well-being (9).

The World Health Organization (WHO) recommends that children and adolescents aged 5–17 years engage in at least an average of 60 min per day of moderate to vigorous-intensity, mostly aerobic, PA across the week (10). According to a recent study on school-attending adolescents, 81.0% of adolescents aged 11–17 years are insufficiently active (not meeting current daily PA recommendations) (11). The 2020 study by Guthold et al. revealed that a higher prevalence of physical inactivity was found amongst girls in almost all countries globally (84.7%, compared with a prevalence of 77.6% for boys), with no significant changes for girls in these trends over the past decade (11).

Persistent low PA levels among adolescent girls constitute a public health concern that calls for immediate and evidence-based policy action. Increasing PA participation among adolescent girls is an important component not just because of the positive effects on health, but also because PA can play a role in economic development, education, peace-building, and trauma relief in different geographical, cultural, and political contexts (12, 13). Promoting PA contributes directly to attain many of the 2030 Sustainable Development Goals (SDG), by promoting policy actions that foster peace, tolerance, respect, and the empowerment of women (14).

Several reviews have investigated the factors that influence PA behavior in adolescent girls and highlighted complex influential factors. For example, a review of the effectiveness of school-based PA interventions on adolescent girls discovered only a small effect of these interventions, namely multicomponent interventions, on increasing PA levels due to complex social and cultural norms that play in the structures of school PA programs rendering limited flexibility in accommodating for gender differences (15). Furthermore, a review of qualitative studies on the self-perception of PA of adolescent girls identified perceived gender bias in sports, along with a limited level of competence in abilities, preference in other priorities, and differing social expectations as impediments for PA participation among adolescent girls (16). Despite the sizable evidence providing insights on the influences of PA behavior for this group, challenges continue to exist in implementing gender-responsive PA interventions (17) leaving girls enduring inequalities in access to and experiences with PA (18).

Limited research has been able to explain how policymakers and implementers can effectively cater toward adolescent girls' needs when it comes to increasing PA levels (11, 16, 19). Common theories used to explain the factors that affect the PA of adolescent girls have used the socioecological model (18, 20, 21). Bronfenbrenner's ecological model of human development describes factors that influence behavior to be linked to individual, interpersonal, and environmental levels (22). Nonetheless, previous studies lack on how to translate these factors into practice in terms of a gender-responsive approach to PA promotion in adolescent girls.

A more gender-responsive approach to PA promotion has been recommended in important policies and strategies. The United Nations and WHO have called on the Member States to act to increase gender equity in PA and sport participation. For example, Article 10 of the Convention on the Elimination of All Forms of Discrimination against Women calls on countries to ensure women have equal opportunities for participation in sport and PA (23), also contributing to achieving the SDG number five, gender equality (24). The WHO, through the Global Action Plan on Physical Activity 2018–2030 (12), also reinforces and supports gender equality and the empowerment of girls to uphold this fundamental human right, and a necessary foundation for a peaceful, prosperous, and sustainable world (24). Accordingly, actions are necessary to tackle inequalities in PA, as reflected by the Physical Activity Strategy for the WHO European Region 2016–2025 (25), by improving the availability, affordability, and acceptability of PA in some age groups. In particular, early adolescence may be an opportune transitional period to promote the development of healthy behavior and equitable gender norms early in life that can be transformative both immediately and over the life course (26, 27).

The gender disparity of PA in adolescents calls for increased efforts at the global, national, and regional levels to reduce inequalities in opportunities for PAs of adolescent girls. Providing a synthesis of the most recent evidence may assist in the development of gender-responsive recommendations, strategies, initiatives, and policies for implementers. Therefore, this systematic review (SR) of systematic reviews (SRs) aims to (i) summarize evidence from SRs examining the barriers and facilitators of PA participation in adolescent girls and (ii) identify key areas for action and policy implementation.

## Methods

An adapted Preferred reporting item for systematic reviews and meta-analysis (PRISMA) reporting method was used to conduct this SRof SRs (28). The PRISMA checklist is available in Supplementary Material 1. The protocol for this review is registered in PROSPERO (reference number CRD42020204023).

### Eligibility Criteria

The study eligibility criteria consisted of the type of studies (SRs and/or meta-analysis); type of participants [adolescent girls 10–19 years of age (according to WHO), or in this age range]; type of interventions (any form of PA practice); and type of outcome (barriers and/or facilitators. Studies were excluded according to study type (editorials, comments, case reports, guidelines, conference abstracts, other reviews); studies without measurement of the outcomes of interest, i.e., studies without barriers or facilitators; studies that aim to find correlations between PA and other factors; studies that aim to analyze the effectiveness of PA interventions; populations with specific conditions (e.g., pregnancy); and studies that do not provide separate data for sex or age category.

### Information Sources

A comprehensive search for relevant SRs was conducted independently by the first and second author until February 2021 using two electronic databases: PubMed and Cochrane Library.

### Search Strategy

The key search terms used in each database were as follows: (“physical activity” OR exercise OR sport^*^ OR “physical education”) AND (child^*^ OR adolescent^*^ OR teen^*^ OR youth OR young) AND (female OR girl^*^ OR women) AND (“gender gap” OR “gender differences” OR factor^*^ OR motive^*^ OR barrier^*^ OR facilitator^*^ OR perception^*^ OR support). We limited the search to study type (SRs, meta-analysis, and reviews) and the English language. The complete search strategy for each database can be consulted in Supplementary Material 2.

### Selection Process

The search results were retrieved and screened from databases by two independent authors according to predefined steps. First, articles were screened by the information from the title and abstract. Second, potentially relevant articles were retrieved for full-text review and their eligibility for the study was determined. Disagreements were resolved through discussion until consensus.

### Data Collection Process

Each selected SR was independently evaluated by the two authors to extract information regarding study design, participants, number and type of included studies, outcome measures, the setting of intervention, and barriers and facilitators. If there were discrepancies in data extraction, authors discussed until consensus.

### Data Items

Barriers and facilitators were identified and then synthesized in three main levels (individual, interpersonal, and environment) from Bronfenbrenner's ecological model of human development (22). The individual level refers to perceptions and attitudes of the individual; the interpersonal level refers to the relations with factors that have direct contact with the individual in their immediate environment, such as parents, siblings, teachers, and school peers; and the environment level incorporates formal and informal social structures, and cultural norms, which do not themselves contain the individual, but indirectly influence them.

### Methodological Quality

Each eligible SR was evaluated regarding its methodological quality by two independent authors, using the 16-item AMSTAR 2 tool (29). Each study was classified according to critical domains that can affect the validity and conclusion of the review. The critical domains considered for this review were: protocol registered before the commencement of the review (item 2); adequacy of the literature search (item 4); justification for excluding individual studies (item 7); risk of bias from individual studies being included in the review (item 9); appropriateness of meta-analytical methods (item 11); consideration of the risk of bias when interpreting the results of the review (item 13); and assessment of the presence and likely impact of publication bias (item 15). The studies were rated as “high-quality” if there were none or there was one non-critical weakness; “moderate-quality” if more than one non-critical weakness; “low-quality” if one critical flaw with or without non-critical weaknesses; and “critically low-quality” if more than one critical flaw with or without non-critical weaknesses. Any disagreements in the classification were resolved by discussion.

## Results

### Study Selection

**Figure 1:**
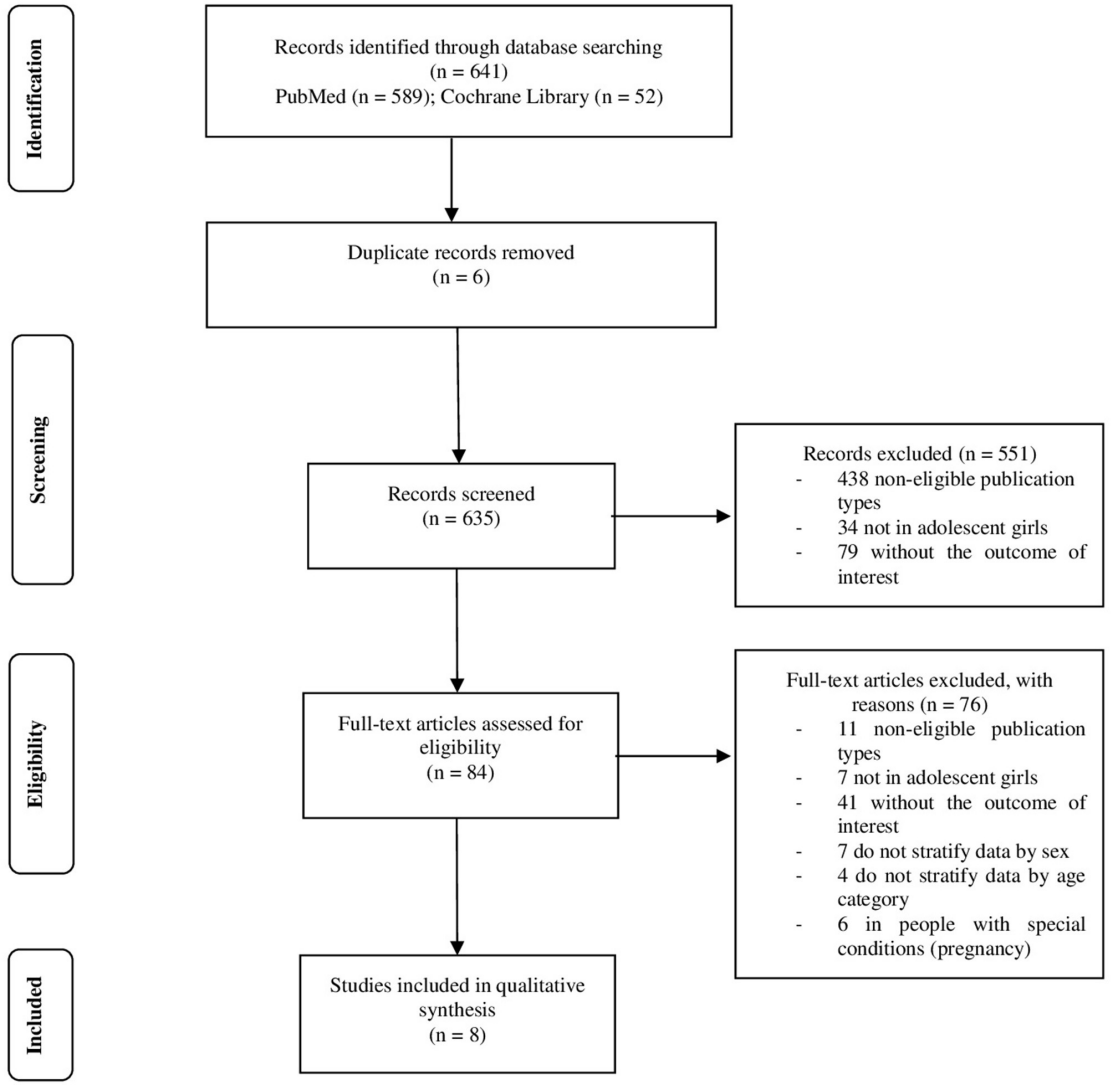
Flow diagram of included studies.

Out of the 641 references identified in the initial search in electronic databases, the study selection process resulted in the inclusion of a total of eight studies in the qualitative synthesis (Figure 1).

### Study Characteristics

The study characteristics included in this review are shown in Table 1. All studies were SRs, ranging from 2006 to 2019, and published in the English language. Concerning the study design of the original studies, the majority provided qualitative designs (16, 31, 33, 35, 36). Regarding the participants, the studies included children and adolescents (30, 31, 34) or adolescents only (16, 32, 33, 35, 36) of the female sex (16, 32, 35) or both sexes (30, 31, 33, 34, 36), with ages ranging from 3 to 25 years. All the SRs included school setting, with some reviews also including community (i.e., public spaces like parks) (16, 31, 32, 36), primary care clinic/health centers (16, 31, 35), and after school hours setting (i.e., organized by school clubs, in or out of the school facilities) (33).

**Table 1:** Characteristics of the included studies.

**References**	**Study type**	**Included studies**		**Participants type**		**Outcomes**	**Setting**
		**Number**	**Design**	**Sex**	**Age range (years)**		
Al-Hazzaa (30)	Systematic review	42	Cross-Sectional	M/F	3–19	Prevalence, correlates, and barriers to PA participation	School
Allender et al. (31)	Systematic review	24	Qualitative	M/F	5–24	Barriers and motivations to PA participation	School, community, general physician referral schemes, sports and leisure clubs, sports governing bodies
Allison et al. (32)	Systematic review	4	Intervention, qualitative	F	11–25	Effectiveness of interventions on team sport participation and PA outcomes in girls	School, community
Corr et al. (16)	Systematic review	24	Qualitative	F	12–18	Perceptions of adolescent girls' PA participation	School, community, health centers
Martins et al. (33)	Systematic review	12	Qualitative	M/F	13–18	Perspectives of adolescents regarding facilitators and barriers to PA participation	School, after school
Rees et al. (34)	Systematic review	16	RCT, CT	M/F	9–16	Barriers and facilitators of PA among young people	School
Standiford (35)	Systematic review	19	Qualitative	F	11–19	Factors that influence PA participation in adolescent girls	School, health centers
Stankov et al. (36)	Systematic review	15	Qualitative	M/F	10–20	Experiences and barriers to PA participation in adolescents	School, community, home

*CT, controlled trial; F, female; M, male; PA, physical activity; RCT, randomized controlled trial*.

### Methodological Quality

Table 2 shows the assessment of methodological quality for each study. All studies were rated with “critically low-quality.” The critical domains where studies did not meet the quality requirements more frequently were the registration of the protocol before the commencement of the review (item 2, *n* = 7) and the consideration of the risk of bias when interpreting the results of the review (item 13, *n* = 6). In non-critical domains, all the reviews did not report the complete population, intervention, control group, and outcome (PICO) components (item 1, *n* = 8) and they also did not report the sources of funding of individual studies (item 10, *n* = 8). The reviewers were in total agreement on the methodological quality assigned to each study.

**Table 2:** Assessment of methodological quality of the selected systematic reviews using AMSTAR 2 tool.

**References**	**Item**															
	**1**	**2**	**3**	**4**	**5**	**6**	**7**	**8**	**9**	**10**	**11**	**12**	**13**	**14**	**15**	**16**
Al-Hazzaa (30)	No	No	No	Partial yes	No	No	Partial yes	No	No	No	No meta-analysis conducted	No meta-analysis conducted	No	No	No meta-analysis conducted	No
Allender et al. (31)	No	No	No	Yes	No	Yes	No	No	No	No	No meta-analysis conducted	No meta-analysis conducted	No	No	No meta-analysis conducted	Yes
Allison et al. (32)	No	No	Yes	Yes	No	No	Partial yes	No	Yes	No	No meta-analysis conducted	No meta-analysis conducted	No	No	No meta-analysis conducted	Yes
Corr et al. (16)	No	Partial yes	Yes	Partial yes	Yes	Yes	Partial yes	Yes	Yes	No	No meta-analysis conducted	No meta-analysis conducted	No	Yes	No meta-analysis conducted	Yes
Martins et al. (33)	No	No	No	Partial yes	Yes	Yes	Partial yes	Partial yes	Yes	No	No meta-analysis conducted	No meta-analysis conducted	No	Yes	No meta-analysis conducted	Yes
Rees et al. (34)	No	No	No	Partial yes	No	Yes	Partial yes	No	Yes	No	No meta-analysis conducted	No meta-analysis conducted	Yes	No	No meta-analysis conducted	Yes
Standiford (35)	No	No	No	Partial yes	No	No	No	Partial yes	No	No	No meta-analysis conducted	No meta-analysis conducted	No	No	No meta-analysis conducted	No
Stankov et al. (36)	No	No	Yes	Partial yes	Yes	Yes	Partial yes	Yes	Yes	No	No meta-analysis conducted	No meta-analysis conducted	Yes	Yes	No meta-analysis conducted	Yes

### Barriers and Facilitators

The barriers and facilitators that seem to have influenced PA participation among adolescent girls are described in Table 3. All the studies provided barriers, and seven studies also provided facilitators (16, 30–35). The most frequent PA barriers were the lack of support from peers (16, 32, 33, 35, 36), family (16, 33–36), and teachers (16, 31–33, 35), followed by lack of time (16, 30, 32, 33, 35). The most identified facilitators for PA were weight loss or management (30–34) and support from the peer (16, 31, 33–35), family (16, 31, 33–35), and teachers (16, 31–33, 35).

**Table 3:** Barriers and facilitators identified in the selected systematic reviews.

**References**	**Barriers**	**Facilitators**
Al-Hazzaa (30)	No appropriate place for PA; lack of time, facilities, and resources	Weight loss
Allender et al. (31)	Negative experiences at school; peer pressure; identity conflict; PE uniforms; boys' dominance in class; competitive classes; lack of teachers support; competitive sports; highly structured activities	Body shape; weight management; beauty; new social networks; family, peer and school support; a safe environment; experimentation; unusual activities
Allison et al. (32)	Family responsibilities; lack of skills; preference for other activities; lack of time; the cost of activities; lack of support from PE teachers	Being healthy; losing weight; looking better; enjoyment atmosphere of sessions; the importance of the coach in enjoyment, motivation, attendance and participatory style of coaching; experimentation of new modalities; opportunity to engage in girls-only activities; provision of clubs/sports; consultation with girls about sports preferences and formats of delivery
Corr et al. (16)	**Gender bias in sport:** Body image/body-centered issues (low perceived competence, high weight status, biological changes during puberty); societal pressure (bullying, stereotypes of femininity, PA contradicts the image of femininity); peer and teachers' feedback (PE teacher not supportive) **Motivation and perceived competence:** Low perceived skill (ability levels and competition); PA opportunities (lack of variety and emphasis on skill) **Competing priorities during adolescence:** Schoolwork and home responsibilities (lack of time due to increased workloads in school, the pressure of parents to perform well at school, increased responsibilities at home); parental expectations (encouragement for different subjects, focus on achieving high grades, working part-time); changes in leisure activities (preference for other leisure activities) **Meeting societal expectations:** Peer influence (friends do not participate in PA, fear of ridicule from friends); adult influence (lack of support and encouragement from teachers or parents); community influence (rural settings are limited in the choice of activities, high costs of membership in clubs, competition)	**Gender bias in sport:** Peer and teachers' feedback (encouragement from teachers for active girls with high skill levels and those who perceive competence) **Motivation and perceived competence:** Perceived skills (high ability levels and competition with friends); PA opportunities (single-sex classes, classes focused on fun and involvement) **Competing priorities during adolescence:** Changes in leisure activities (increased opportunities with older ages) **Meeting societal expectations:** Peer influence (friends participate in PA); adult influence (perceived support and encouragement from teachers or parents); community influence (variety of activities in urban settings)
Martins et al. (33)	**PA attitude:** Competition climate; negative experiences in PE classes; the pressure of winning and failing in front of their peers and peers' negative reactions; not feeling comfortable, absence of fun, and learning opportunities **Motivation:** Lack of motivation and enthusiasm **Fun:** Lack of fun **Perception of competence:** low perception of competence **Body image and exposure concerns:** Feeling discomfort in front of others and physical appearance (sportswear, sweating, weight) **Perception of femininity and social norms:** Femininity is not compatible with PA **Time and competing for leisure activities of PA:** Lack of time; preference for other leisure activities **Influence of friends, family, and significant others:** Preference for other activities from friends; negative experiences with friends; lack of support of friends; physically inactive family members; lack of encouragement, financial support, and transportation from family; too much pressure to improve academic performance; sessions focused on competition and not inclusion, not fun-oriented, high levels of intensity; lack of encouragement from teachers; curriculum focused on sports perceived as masculine **Environmental opportunities:** Lack of offers of PA programs in school and community; difficulty	**PA attitude:** Active girls associate with health benefits, physical appearance, social interactions, positive experiences, fun, diversity of activities **Fun:** Having fun **Perception of competence:** High levels of perception of competence **Body image and exposure concerns:** Good appearance and adequate weight **Perception of femininity and social norms:** Media in changed social norms **Time and competing leisure activities of PA:** PA opportunities during school time **Influence of friends, family, and significant others:** Presence and practice of PA with friends; friends support; encouragement, transportation, financial support, Support of PA from family; PA with their family members; participation in sports clubs as a child; encouragement, support, help in improvement in activities and explanation of PA benefits from PE teachers; the presence of adults close to the PA locations for security reasons **Environmental opportunities:** Opportunities at school (PE, school sports, break time, field trips); neighborhood facilities, low cost of PA programs; the existence of facilities and equipment in school, at home and in the neighborhood
	accessing PA programs (costs, time, schedule, distance from home, lack of transportation, and low security); lack of infrastructures **Life transition periods:** Adolescents with low PA levels in the transition from primary to high school, due to increased workload, lack of motivation, fewer opportunities, focus on competition, and giving more importance to other activities	
Rees et al. (34)	**PA and school:** PE environment and rules **PA and family and friends:** Parental constraints (safety, monitoring leisure time to ensure that have time to do homework and domestic chores, disapproval of exercise) **Peer constraints:** Prejudiced attitudes of boys **PA and the self:** Feelings of discomfort during PA; self-consciousness about bodies/appearance **PA and practical and material resources:** Provision of highly structured activities	**PA and school:** Consultation in choice of activities; mixed-sex activities and the chance to participate in activities traditionally seen as being for young men **PA and family and friends:** Parental support (encouragement and material resources); chance to make new friends **Peer constraints:** Social support from friends **PA and the self:** Help with losing weight; articles promoting PA in magazines
Standiford (35)	**Perceptual influences:** Appearance concerns (maintaining a feminine physical appearance); personal barriers to PA (preference for sedentary activities, lack of time, lack of motivation, perception PA is not fun, lack of money for sports, and transportation to gyms); body image (discomfort at doing PA in front of others in PE classes/gym, being objectified by boys in PE classes or active play, being bullied or harassed due to the body size and shape) **Interpersonal influences:** Ability comparison and competition (poor performance, conflict with classmates, fear of criticism in PA, lack of PA choices in PE for those who are less physically fit); family, peer, and teachers influence (safety concerns, belief that PA is not proper for girls, time constraints; intimidation, bullying, and exclusion in PE classes); contending with boys **Situational influences:** Accessibility and availability (preferred sport not available, community sports clubs expensive, school sports programs have outdated equipment and inconvenient hours, distance from recreational centers for rural girls); PA and gender role (social pressure of appearance and behavior, fewer sports opportunities than boys); safety concerns (perception of dangerous or crime in the environment of recreational centers and neighborhood outdoor areas)	**Perceptual influences:** Perceived benefits of PA (positive beliefs about engaging in PA: stay in shape, looking good, feel better, relaxed and energetic, health risk reduction, more fun than sedentary pursuits, a good time to be alone, escape the pressure, and stresses of everyday life); PA enjoyment (feel of enjoyment, being able to choose activities without control from others); favorite PA (possibility for choosing preferred activities and its delivery) **Interpersonal influences:** Ability comparison and competition (good performance); family, peer, and teachers influence (rides to the gym, monetary support for sports; opportunity to socialize). **Situational influences:** Accessibility and availability (geographical proximity and social and financial feasibility of PA programs)
Stankov et al. (36)	**Environment:** Regulatory environment (inappropriate teaching practices, having to wear a uniform or follow a dress code during PE classes, promotion of sedentary activities); built environment (lack of privacy in changing rooms, lack of resources, lack of neighborhood safety); inhibitory social norms (the perception that PA is not culturally valued); physical environment (weather conditions) **Interpersonal:** Victimization (verbal and physical bullying, social exclusion, and stereotyping by peers); nature of household (noisy and cramped household); lack of social support (peers and family) **Individual:** Negative body image (perception of not looking good as others during PE classes); perceived victimization (perception of being verbally bullied by peers during PE classes); perceived inferiority in social settings (perception of being negatively judged by others during PE classes, perception of reduced ability relative to others, dislike of being visible in PE classes); competing demands on time	NR
	(obligations to family and friends, school and homework commitments, length of travel to and from school for the ones in rural areas); lack of motivation (persistent failure during PA, lack of motivation by laziness); physical factors (physical discomforts, too out of shape, fatigue after the day and exercise, perceived beauty cost); capacity (lack of competence in setting goals, low self-efficacy or confidence in the ability to seek support given problems with transportation); lack of knowledge (false beliefs about PA, the perception that PA is of little benefit)	

*NR, not reported; PA, physical activity; PE, physical education*.

#### Individual

The most commonly mentioned barriers at the individual level were lack of time (16, 30, 32, 33, 35), lack of perceived competence (16, 32, 33, 36), discomfort during and after PA (33–36), increased obligations and responsibilities toward family and friends (16, 32, 34, 36), and preference for other leisure activities (16, 32, 33, 35). Regarding the facilitators for PA, the results show that the most reported in the individual level were weight loss or management (30–34), looking good and beauty perceptions (31–33, 35), perceived competencies (16, 33, 35), benefits of PA (33, 35), and enjoyment (33, 35).

#### Interpersonal

The most commonly mentioned barriers at the interpersonal level were the lack of support from family (16, 33–36), peers (16, 32, 33, 35, 36), and teachers (16, 31–33, 35). The most commonly mentioned facilitators at the interpersonal level were family (16, 31, 33–35), peer (16, 31, 33–35), and teachers (16, 31–33, 35), and the opportunity to socialize (31, 33–35).

#### Environmental

The most commonly mentioned barriers at the environmental level were the costs of activities available (16, 32, 33, 35), and safety concerns of the neighborhood outdoor areas and environment (33, 35, 36). The most commonly mentioned facilitators in the environmental level were the accessibility and availability of recreational centers (16, 32, 35), the opportunity to explore new activities (31, 32, 35), the consultation with girls about sports preferences and formats of delivery (32, 34), and the role of media have on influencing change in social norms (33, 34).

The distribution of the barriers and facilitators among the three levels (individual, interpersonal, and environment) is presented in Table 4.

**Table 4:** Summary of barriers and facilitators in the selected systematic reviews.

**Barriers**	**Facilitators**
**Individual**	**Individual**
Perception of not looking good as others in PE classes (16, 36)	Looking good/beauty (31–33, 35)
Physical appearance (sportswear, sweating, weight) (33, 34)	Weight loss or management (30–34)
Lack of perceived competence/capacity/skills (16, 32, 33, 36)	Perceived competence/capacity/skills (16, 33, 35)
Lack of knowledge about PA benefits (36)	Perceived benefits of PA (33, 35)
Lack of time (16, 30, 32, 33, 35)	Good time to be alone (35)
Perception that PA is not fun (33, 35)	Feel of enjoyment/ having fun (33, 35)
Discomfort during and after PA (33–36)	Feel better, relaxed and energetic (35)
Increased obligations and responsibilities to family and friends (16, 32, 34, 36)	Escape the pressure and stress of everyday life (35)
Preference for other leisure activities (16, 32, 33, 35)	Positive experiences of PA practice (33)
Perception of being bullied in PE classes (35, 36)	Being healthy (32)
Increased school and homework commitments (16, 36)	Stay in shape (31, 35)
Working in part-time (16)	Health risk reduction (35)
Perception of inferiority in PE classes (36)	
Fear of failing in front of their peers (33)	
Lack of motivation (33, 35, 36)	
**Interpersonal**	**Interpersonal**
Verbal and physical bullying by peers (35, 36)	Opportunity to socialize (31, 33–35)
Social exclusion by peers (36)	Enjoyment atmosphere in PE classes (16, 32)
Lack of peer support (16, 32, 33, 35, 36)	Peer support (16, 31, 33–35)
Lack of family support (16, 33–36)	Family support (transportation, financial support, encouragement) (16, 31, 33–35)
Lack of teachers support (16, 31–33, 35)	Teachers support (16, 31–33, 35)
Competitive environment in PE classes (31, 33)	Competition with friends (16)
Friends do not engage in PA (16, 33)	PA practice with friends (33)
Family members do not engage in PA (33)	PA practice with family members (33)
Poor performance in PE classes (35)	
Boys' dominance in classes (31)	
Prejudiced attitudes of boys (34)	
Negative experiences with friends (33)	
Negative experiences at school (31, 33)	
Lack of PA choices in PE classes (16, 35)	
Highly structured activities at PE classes (31, 34)	
Parents encouragement for different subjects (16)	
Parents' focus on achieving high grades (16, 33)	
Lack of financial support and transportation to gyms (33, 35)	
Adverse household conditions (36)	
Stereotyping by peers (16, 36)	
**Environment**	**Environment**
Promotion of sedentary activities (36)	Consultation with girls about sports preferences and formats of delivery (32, 34)
Perception that PA is not culturally valued (16, 36)	Media in the change of social norms (33, 34)
Social pressure of feminine appearance and behavior (16, 35)	Opportunity to engage in girls-only activities (16, 32)
Safety concerns at neighborhood outdoor areas and environment (33, 35, 36)	Safe environment (31)
Limited access to sports/programs in the community (clubs, preferred sports) (33, 35)	Accessibility and availability of recreational centers/clubs/activities (16, 32, 35)
Limited activities choices in rural settings (16)	Possibility of choosing/experiment activities and its delivery (31, 32, 35)
Expensive activities (16, 32, 33, 35)	Low cost of programs (33)
High distance from recreational centers at community (33, 35)	Geographic proximity (35)
Lack of facilities (30, 33)	School, neighborhood, and home facilities (33)
Fewer opportunities than boys (35)	Opportunity to engage in mixed-sex activities or in activities traditionally seen as being for men (34)
Limited access to sports/programs in school (33)	PA opportunities during school time (33)
Lack of transports (33)	
Lack of resources (30, 36)	
Inappropriate teaching practices (36)	
Curriculum focused on sports perceived as masculine (33)	
Outdated sports equipment at school (35)	
Having to wear a uniform in PE classes (31, 36)	
Lack of privacy in changing rooms (36)	
Biological changes during puberty (16)	
Life transition periods (33)	
Weather conditions (36)	
No appropriate place for PA practice (30)	

*PA, physical activity; PE, physical education*.

## Discussion

### Summary of Main Findings

This SR of SRs summarizes the evidence of eight studies examining the barriers and facilitators of PA in adolescent girls. This synthesis has highlighted that the most frequent PA barriers reported in adolescent girls were found to be the lack of support from peers, family, and teachers, followed by the lack of time; the most identified facilitators reported for the PA of adolescent girls were weight loss/management, and peer, family, and teachers support (16, 31–33, 35). The majority of reported factors of influence hindering and/or supporting PA gathered in this review were mainly located in the individual and interpersonal levels.

### Barriers and Facilitators

The socio-ecological model is one approach commonly mentioned as the most suitable method to illustrate complexities of examining the individual, physical, and social environmental factors when explaining PA behavior in general, therefore can be viewed as applicable for illustrating PA behavior of adolescent girls. Although the data were aggregated according to the three main levels, it is essential to note that there is a dynamic cross-over of barriers and facilitators, as one facilitator and/or barrier may be linked to another on a different level. For example, limited social support by a coach/teacher may occur due to the limited physical ability/skill of an individual leading to an overall negative experience, as mentioned in one review (35).

Although the barriers and facilitators by level can be seen separately, it is important to acknowledge the interplay and complexity of each and how they affect the participation of adolescent girls in PA overall when targeting within interventions. Further research is needed in terms of appropriate theoretical frameworks that can provide a clearer way to illustrate such complex intertwined factors that influence the PA participation of adolescent girls. This, in turn, can provide improved guidance when designing multicomponent interventions to target multiple influential factors that address these complex factors affecting PA participation of adolescent girls.

Approaches that can transform the role and relationship of harmful gender norms should be an aim of PA promotion (37). Gender norms have been known to play a significant role not only in the diverse exposure levels to the risk factors of non-communicable diseases in men and women but also in the access to and the use of services and resources (37). Gender norms and stereotypes have a specific influence on PA participation by creating societal expectations of what it means to be feminine/masculine that may limit the access to and use of PA opportunities available. For adolescent girls, gender stereotypes are seen as a barrier toward PA participation. With PA opportunities seen as socially appropriate to boys only, a perceived gender dominance occurs, which decreases the value and access to PA opportunities for girls (16, 30, 33, 35, 36). Not only do these norms hinder access to opportunities, but they also play a significant role in body image concerns. Although, body image was perceived as a facilitator in this synthesis (30–35) warrants should be made regarding the potential to negatively reinforce gender stereotypes that could be harmful to the physical and mental health of girls. Additionally, challenges exist in implementing gender-responsive PA interventions aimed at adolescent girls, calling for increased research and mechanisms on how to effectively respond (17).

#### Individual

Examples of individual barriers common across the reviews were associated with body image perceptions, such as feeling uncomfortable while participating in PA due to the concerns of appearance and discomfort that for example, were linked to the type of clothing worn and/or perceived skill level (16, 31–36). Consistent with previous research, negative body image and dissatisfaction have been associated with the age of adolescent girls which can contribute to the negative perceptions and experiences around PA participation (18, 38). Interventions that can increase the perceived competencies of girls could be considered one way of which to target negative self-ideals such as PA opportunities that focus on individual progression (18). However, self-perception has been considered largely influenced by the interpersonal and greater social contexts of cultural/gender ideals reinforced by family and peer groups (39). Evidence has suggested that mothers' conversation with adolescent girls around positive body image to be one way to foster improved body satisfaction (40), which may also aid in improved experiences with PA participation.

Adolescence is often marked as a highly sensitive and complex period of transition from childhood to adulthood (16). Further heightening vulnerabilities, biological changes experienced by adolescent girls during puberty can lead to discomfort while menstruating and combined with a heightened sense of self-consciousness which may, in fact, discourage participation in this age group (16). Research has suggested early maturing girls to be particularly prone to develop negative body images as they experience contradictions to the cultural ideals placed on them (39). Limited research has explored the role menstruation may play in hindering PA participation of adolescent girls since only one review in this synthesis mentioned menstruation as a barrier (16). Thus, further research should explore mechanisms to combat potential barriers of menstruation around PA participation as this review found limited evidence suggesting such implications.

#### Interpersonal

Interpersonal facilitators and barriers most commonly reported in this SR were the variety of and/or lack of support providers for PA of adolescent girls (16, 31–35). Support by peers, family, and teachers was mentioned evenly amongst the included reviews in this synthesis, potentially suggesting that each provider of support may have an equivalent influence on the PA of girls. A qualitative study that attempted to provide mechanisms for social support in PA for adolescents observed that receiving reinforcement and praise for PA, providing opportunities to practice and improve performance, and providing the practical support necessary to participate (equipment/money/transport) were key forms of support (41).

It is important to note that each support provider may play a different role in influencing the participation of girls in different settings. For example, supportive parents were found in this synthesis to play an essential role for adolescent girls by promoting PA time outside of school through means, such as financial support for the activities, transportation to activities, or overall encouragement to participate (16, 31, 33, 34). Similar research has acknowledged the influential support by parents toward PA of adolescent girls, especially young adolescents who may be more reliant on parents' ability to provide access to PA opportunities (i.e., equipment, cost of activity, and transportation) (42, 43).

This form of support places a burden on the financial ability and time of parents to provide the necessary access to resources and transportation to PA facilities, which may foster the potential inequalities from socioeconomic classes unable to provide the financial support required for adolescent girls to participate. Such disparities can also be seen within ethnic/minority PA participation of adolescent girls (18, 44, 45). Other forms of social support may provide a greater influence in PA participation less contingent on the family. For instance, social support by friends discovered in previous research was to be a greater predictor of meeting the daily PA recommendations for those with low access to PA resources (43). Teacher support, especially important in terms of school PA participation, influences adolescent girls from this synthesis mirrors existing research as well. Evidence has suggested that teacher support in the form of providing encouragement, praise, feedback, and enthusiasm directly targeting adolescent girls can provide increased positive dialog and engagement, which has been reportedly inexperienced by this group (41, 42).

#### Environmental

Most of the SRs in this synthesis focused on the school setting PA and reported barriers across all three levels: be it the teachers, social or physical environment, or the activity/curriculum. While numerous efforts have been made in the past decade to increase PA and sport participation of adolescent girls in this setting, many interventions have been found to have only small to limited effects (15). This may be due to the multidimensional barriers that span across the three levels, making it difficult to accurately address existing interventions targeting adolescent girls. Despite this, schools may be an important entry point for improved PA promotion among girls, especially if they are effective in changing the traditional structure of PE that hinders participation. This is consistent with existing literature that has made claims of gender biases in the PE curriculum calling for new ways of developing a more inclusive approach (46).

Considerations should be made to the social and built environment of the setting that offers PA in schools to be appropriate to gender preferences and needs. For instance, this synthesis found that some girls may prefer enjoyable activities that are non-skill-based, provide social opportunities, along with having a variety of activities to choose from, and the autonomy of which activity to participate in (16, 31–35). Alternatives to competitive sports with a focus on physical fitness and enjoyment could help adolescent girls develop positive PA habits according to their abilities and skills, which could, in turn, increase the likelihood of lifelong engagement in PA (16, 33).

Environments outside of the school setting, such as the constructed setting of parks, sidewalks, street lights, distance to school, and the neighborhood that one lives in have found an influence on the PA levels in adolescent girls (42, 47, 48), which is in accordance with findings from this review.

### Limitations

Notwithstanding, there are limitations that must be considered when interpreting the findings from this synthesis. Although this SR aimed to identify key barriers and facilitators for adolescent girls on a global level, we acknowledge that adolescent girls may face different obstacles unique to their social, cultural, and regional contexts that cannot be generalized in this review.

A major gap highlighted in this review is that most of the SRs included studies that were primarily conducted in high-income settings. As the global disparities of PA continue to rise in adolescent girls, additional interventions and research is needed throughout diverse regional settings due to the potential contextual variances in PA norms and environmental situations (15, 16, 49).

Despite that general implications can continue to be warranted to an extent, it is important to also note that differences in perceptions of barriers and facilitators in adolescent girls could relate to how active or inactive a girl is, as some evidence discovered not all girls prefer the same type of activities (16, 33). In addition, some barriers and facilitators explicitly mentioned for adolescent girls in this review may also affect adolescent boys to some extent, given the adolescent period of social pressures and norms.

One limitation worth noting is that the SRs included in this review did not exclusively reflect adolescent girls aged 10–19, as some reviews reported on both sex and a variety of age ranges. Therefore, some barriers and facilitators may not be representative for adolescence, but also for transition periods, such as childhood and early adulthood. By including studies in both sexes, it allowed us to highlight the limitation that barriers and facilitators mentioned in this review may not only affect adolescent girls, thereby highlighting the difficulties placed in providing a clear gender-responsive direction in targeting interventions. For example, SRs included in this study that investigated both genders identified a few common barriers across genders, such as lack of time (30, 33), perceived skill level (33, 36), and family support (33, 34, 36).

Furthermore, included SRs were ranked as having low methodological quality. However, besides the AMSTAR 2 Tool being described as adequate for the assessment of the methodological quality of SR of SRs, this tool is more appropriate for healthcare interventions and not for studies of a qualitative nature. Therefore, results should be interpreted with caution.

### Implications for Action

The findings of this review aimed to provide a backdrop of considerations for policymakers and implementers when taking a gender-responsive approach to the promotion of PA in adolescent girls. Based on the evidence synthesized in this review, key courses of action can be applied in practice.

#### Inclusive Approach to Curriculum Development to Address Gender Norms

It is necessary to have an inclusive approach to curriculum development for adolescent girls to address their specific needs. An Intersectoral approach involving education, sports, and health sectors should be considered in designing PE curricula that place a gender-responsive view of preferences of adolescent girls. This may require policies within the education and sports sector that can hold accountable the gender-responsive targeted action required in these modifications.

More dialog addressing harmful gender norms in the settings where PA takes place may help identify and address the barriers that discourage participation (33). Examples may include involving institutions, teachers, and boys in recognizing and addressing their own contribution to enabling these norms and encourage fostering a supportive environment for PA participation of girls. A curriculum that is non-competitive, flexible, individually created with a variety of opportunities, and the time to perform PA are some examples of how to adapt to the preferences of adolescent girls. Policies and initiatives that can support PA outside the traditional PE setting in schools, such as active breaks and active extracurricular activities are additional ways to encourage increase PA opportunities.

#### Adequate Training of Professionals to Ensure Inclusion of Adolescent Girls

Adequate training of professionals is required to equip them with the knowledge and skills to provide an inclusive PA environment for adolescent girls. Trained professionals should possess the understanding of the negative implications of lack of support provided to PA of girls, regardless of skill level and strategies on how to be more gender-responsive in their support provided specifically to girls. Qualified professionals should consider focusing on increasing physical literacy at earlier ages to build the confidence and skills required to maintain adequate PA levels throughout adolescence (50). Policies should be in place to ensure continued professional development and the availability of appropriate resources and material to provide gender-responsive PA strategies in school and sports club settings. Strategies and proper training of parents and peers to provide support for the PA participation of girls should also be considered due to its strong influence in facilitating PA (16, 31, 33–35). Examples of ways in which parents and peers can encourage PA support in girls include participating in PA with adolescent girls and providing positive reinforcement for conducting PA (41). Interventions that can provide family participation and engagement in PA should also be considered.

#### Environmental Changes in and Out of Schools to Stimulate Participation, to Allow Adolescent Girls to Be Active in a Safe and Attractive Environment

It is essential to stimulate PA participation with environmental modifications. Increasing access to safe and comfortable physical infrastructures and facilities for the use of adolescent girls should be a priority within and outside the school setting. Cost-effective PA interventions are needed so that they can be delivered with limited resources with little to no cost to families and increasing family support and participation of all children and adolescents, but with special attention toward techniques that can encourage adolescent girls specifically (33).

Policies that aim to provide equal opportunities regardless of gender and financial resources to tackle the larger structural barriers toward PA participation in adolescent girls are required. At the local level, policies and initiatives should address how public open spaces can be rendered more appealing to girls regarding safety and accessibility by addressing their specific concerns.

Community mobilization and engagement are necessary to foster a conducive environment supportive for the participation of adolescent girls in these spaces. Schemes that can encourage active travel to and from school are other ways in which schools and local authorities can work together toward increasing PA opportunities. In addition, recreational PA initiatives that can encourage peer engagement of adolescent girls should also be considered.

#### Multistakeholder Support at the Local, Regional, and National Level in Incorporating a Gender-Responsive Approach Toward PA Participation

For the promotion of PA to be effective, it is essential for multistakeholder engagement at the individual, community, cultural, political, and environmental levels (51). Investing in policies that encourage PA for adolescent girls across these levels in and outside the school setting is deemed necessary for taking a gender-responsive action toward PA promotion. For example, active commuting to and from school has been described as a way to enhance PA practice, but this can be compromised if other measures are not implemented, such as safe sidewalks and cycle paths that provide routes to school along with neighborhood safety (8, 52, 53).

At the country level, leadership and coordination across sectors are fundamental to maximize the response required to address low levels of PA of adolescent girls. Ensuring national measures are taken to promote gender equality in PA can be reflected in policies that support gender-relevant forms of activity, particularly in school and recreational time, which can decrease sedentary leisure time activities (25).

The attainment of effective policies that increase PA levels in adolescent girls will contribute to meeting SDG number five, by promoting gender equality and the empowerment of girls at all levels, both essential for a peaceful, prosperous, and sustainable world (24). Gender equality and norms are strengthened and integrated during the adolescence period, when transitions to individualized identities, sexual activity, labor force participation, and marriage may occur. Interventions in the early stage in patterns of health behaviors, such as PA, will influence health trajectories over the life course (27).

### Future Research

Despite the available evidence on the multiple barriers and facilitators that influence the PA of adolescent girls, research is required in terms of appropriate theoretical frameworks that can provide a clearer way to illustrate the multifaceted factors that influence the PA participation of adolescent girls. Such evidence can guide intervention designs aimed to target the multiple influential factors for PA promotion of adolescent girls and provide a clearer picture of what a gender-responsive approach toward action can look like.

Future research on PA should explore the factors for increasing PA among specific subgroups of adolescent girls (e.g., low socioeconomic status, migrant, transgender, girls living with disability, and pregnant/post-partum adolescent girls) along with the barriers and facilitators of transition periods (childhood to adolescence and adolescence to adulthood) longitudinally, to further promote PA in a life-course approach. Further research should also explore developing and testing gender-specific and gender-transformative PA programs for this population to gain effective and evidence-based PA programs, good practices and policies, at local, regional and national levels, as well as its proper monitoring and evaluation. Such evidence is needed to disseminate practical recommendations from research for policymakers at different levels.

## Conclusion

This SR of SRs highlights the collected evidence that there is a variety of factors that influence the PA of adolescent girls. Lack of support from peers, family, and teachers, and the lack of time were among the most frequent barriers for adolescent girls while the motivation for weight loss and support from peers, family, and teachers were the most frequent facilitators toward PA participation.

This study provided key areas for policy action to promote PA in adolescent girls, based on the most frequent barriers and facilitators experienced. For the attainment of effective policies and practices that increase PA levels in adolescent girls, a multisectoral and multilevel gender-responsive response is necessary.

## Data Availability Statement

The original contributions presented in the study are included in the article/Supplementary Material, further inquiries can be directed to the corresponding author/s.

## Author Contributions

KD, AB, SW, RM, IA, AT, KA-O, PG, and JB: conceptualization and writing—original draft preparation. KD, AB, SW, and RM: methodology. KD, AB, and RM: formal analysis. KD and AB: interpretation of data for the work. SW, RM, IA, AT, KA-O, PG, and JB: writing—review and editing and supervision. All authors have read and agreed to the published version of the manuscript.

## Funding

This work was support by a grant from the Government of the Russian Federation in the context of the WHO European Office for the Prevention and Control of Non-communicable Diseases.

## Author Disclaimer

The authors alone are responsible for the views expressed in this publication, and they do not necessarily represent the views, decisions, or policies of the institutions with which they are affiliated.

## Conflict of Interest

SW, IA, and JB are staff members of the WHO. KD, AB, and RM are WHO consultants. The remaining authors declare that the research was conducted in the absence of any commercial or financial relationships that could be construed as a potential conflict of interest.

## Publisher's Note

All claims expressed in this article are solely those of the authors and do not necessarily represent those of their affiliated organizations, or those of the publisher, the editors and the reviewers. Any product that may be evaluated in this article, or claim that may be made by its manufacturer, is not guaranteed or endorsed by the publisher.
