# Supplementary material for: Barriers and Facilitators of Physical Activity Participation in Adolescent Girls: A Systematic Review of Systematic Reviews
Source: Front Public Health. 2021 Oct 15;9:743935. doi: 10.3389/fpubh.2021.743935 (PMC8553996; doi:10.3389/fpubh.2021.743935)
Supplement: Supplementary file 2 [file Table_2.DOCX]

**Electronic Supplementary Material Appendix S2. Full search strategy**

| **Database** | **Search** | **Limits** |
| --- | --- | --- |
| PubMed | (“physical activity” [Title/Abstract] OR exercise [Title/Abstract] OR sport* [Title/Abstract] OR “physical education” [Title/Abstract]) AND (child* [Title/Abstract] OR adolescent* [Title/Abstract] OR teen* [Title/Abstract] OR youth [Title/Abstract] OR young [Title/Abstract]) AND (female [Title/Abstract] OR girl* [Title/Abstract] OR women [Title/Abstract]) AND (“gender gap” [Title/Abstract] OR “gender differences” [Title/Abstract] OR factor* [Title/Abstract] OR motive* [Title/Abstract] OR barrier* [Title/Abstract] OR facilitator* [Title/Abstract] OR perception* [Title/Abstract] OR support [Title/Abstract]) | systematic review, meta-analysis, review, English language |
| Cochrane Library | [Title Abstract Keyword] “physical activity” OR exercise OR sport* OR “physical education”  AND [Title Abstract Keyword] child* OR adolescent* OR teen* OR youth OR young  AND [Title Abstract Keyword] female OR girl* OR women  AND [Title Abstract Keyword] “gender gap” OR “gender differences” OR factor* OR motive* OR barrier OR facilitator* OR perception* OR support | Cochrane Reviews |
